# The Novel Toll-Like Receptor 2 Agonist SUP3 Enhances Antigen Presentation and T Cell Activation by Dendritic Cells

**DOI:** 10.3389/fimmu.2017.00158

**Published:** 2017-02-21

**Authors:** Xueheng Guo, Ning Wu, Yingli Shang, Xin Liu, Tao Wu, Yifan Zhou, Xin Liu, Jiaoyan Huang, Xuebin Liao, Li Wu

**Affiliations:** ^1^Institute for Immunology, Tsinghua-Peking Center for Life Sciences, Tsinghua University School of Medicine, Beijing, China; ^2^College of Veterinary Medicine, Shandong Agricultural University, Tai’an, Shandong, China; ^3^Collaborative Innovation Center for Diagnosis and Treatment of Infectious Diseases, Tsinghua University School of Pharmaceutical Sciences, Beijing, China

**Keywords:** TLR2 agonist, adjuvant, dendritic cell, antigen presentation, cytotoxic T lymphocyte

## Abstract

Dendritic cells (DCs) are highly specialized antigen-presenting cells that play crucial roles in innate and adaptive immunity. Previous studies suggested that Toll-like receptor (TLR) agonists could be used as potential adjuvants, as activation of TLRs can boost DC-induced immune responses. TLR2 agonists have been shown to enhance DC-mediated immune responses. However, classical TLR2 agonists such as Pam3CSK4 are not stable enough *in vivo*, which limits their clinical applications. In this study, a novel structurally stable TLR2 agonist named SUP3 was designed. Functional analysis showed that SUP3 induced much stronger antitumor response than Pam3CSK4 by promoting cytotoxic T lymphocytes activation *in vivo*. This effect was achieved through the following mechanisms: SUP3 strongly enhanced the ability of antigen cross-presentation by DCs and subsequent T cell activation. SUP3 upregulated the expression of costimulatory molecules on DCs and increased antigen deposition in draining lymph nodes. More interestingly, SUP3 induced less amount of pro-inflammatory cytokine production *in vivo* compared to other TLR agonists such as lipopolysaccharide. Taken together, SUP3 could serve as a novel promising immune adjuvant in vaccine development and immune modulations.

## Introduction

The agonists of Toll-like receptors (TLRs) especially recognized by dendritic cells (DCs) promote activation of innate immunity and help initiate adaptive immune responses. These TLR agonists thus can be applied as immune adjuvants to enhance DC-mediated vaccination and cancer immunotherapy (1, 2). Currently, some TLR agonists, such as TLR4 agonist monophosphoryl lipid A (MPL), have been licensed to be used as adjuvants in vaccination and approved (at least in some countries) in oncological conditions (3–7). TLR2 is a conserved molecule expressed by both murine and human conventional DCs (cDCs), and TLR2 agonists have been shown to have great potential to be utilized as adjuvants to enhance cDC-mediated immune responses (8). However, until now TLR2 agonists have not been approved as clinical adjuvants, possibly due to their poor stabilities and thus weak potency *in vivo* (1, 2, 9). It is therefore desirable to design new TLR2 agonist, if it would be used as an adjuvant in immunotherapy.

Innate immunity represents the first line of defense against invading pathogens. Innate immune cells like DCs sense danger signals and initiate responses against them. As the most efficient antigen-presenting cells (APCs), cDCs are in charge of recognizing antigens and promoting adaptive immunity through activation of naïve T cells (10, 11). Stimulation of DC upon recognition of antigens *via* multiple pattern recognition receptors (PRRs) upregulates the expression of costimulatory molecules and induces the production of various cytokines and chemokines, contributing to T cell activation and inflammatory responses. Therefore, DCs bridge innate and adaptive immunity and play pivotal roles in orchestrating immune responses (12). Murine splenic DCs are heterogeneous populations consisting of CD8^+^ cDCs, CD11b^+^ cDCs, and plasmacytoid DCs (pDCs) (13, 14). CD8^+^ cDCs are specialized in antigen cross-presentation *via* major histocompatibility complex (MHC)-I to activate CD8^+^ T cells and ultimately differentiate them into cytotoxic T lymphocytes (CTLs), suggesting CD8^+^ cDCs as essential initiators of cellular immunity. Moreover, CD8^+^ cDCs can polarize CD4^+^ T cells into Th1 phenotype by direct antigen presentation through MHC-II pathway and by producing IL-12p70. The CD11b^+^ cDCs mainly uptake exogenous antigens and directly present them to CD4^+^ T cells *via* MHC-II to induce differentiation of multiple subsets of helper T cells, including Th2 and Th17, which are crucial in defense against extracellular pathogens like parasites and bacteria (15). In contrast, pDCs are characterized by secreting large amount of type I interferons (IFNs) upon viral infection, but have limited antigen presentation ability. The heterogeneity of DCs highlights the concept of division of labor through functional specialization of DC subsets (15).

Pathogen-associated molecular patterns induce functional maturation of immune cells through binding on specific PRRs and enhancing the cellular functions. For instance, ligation of TLR on DCs effectively program DCs into an active status and dramatically enhance DC functions. Therefore, TLR agonists are widely used as stimulators of DCs and enhancers of DC-mediated immune responses (2, 16, 17). TLR2 is a special member among TLR families that needs to form heterodimers with TLR1 or TLR6 to recognize triacyl (Pam3CSK4) or diacyl (Pam2CSK4) lipopeptides, respectively (18, 19). Cell surface receptor TLR2 recognizes lipopeptides mainly derived from cell wall components of bacteria to elicit innate signaling events. TLR2 represents one of important sensors to pathogens and TLR2 deficiency impairs host defense against bacterial infection (20, 21). As TLR2 is conserved in mouse cDCs and their human counterparts, especially between murine CD8^+^ cDCs and the human equivalent CD141^+^ cDCs (22), agonists targeting TLR2 could be promising adjuvants in designing vaccines through activating DCs for cancer immunotherapy. Previous studies showed that conjugation of antigens and TLR agonists targeting APCs achieved dramatically more potent responses than combination of antigens and TLR agonists (23–26). Unlike other TLR ligands, the intrinsic peptide component of TLR2 ligands provides the possibility to directly conjugate antigenic peptides within TLR2 agonists. Notably, the expression of TLR2 by DCs, providing the potential for using TLR2 agonists as adjuvants in DC-mediated immune responses, and so far increasing number of TLR2 agonists have been developed (24, 27–30).

Recently, it has been reported that TLR2 agonists could be used directly (31–34) or as structurally modified forms (26) in cancer treatment, suggesting that TLR2 agonists can be potential effective enhancers for cancer immunotherapies. Administration of TLR2 agonists could enhance effector and memory T cell responses, leading to elevated efficacy of vaccination and tumor rejection (24, 30, 31, 33, 35). Moreover, TLR2 agonists could sensitize B cell lymphoma to chemotherapeutic agents *via* upregulating costimulatory molecules to increase their sensitivity to NK cell and CTL cytotoxicity (32), or inducing caspase 8-dependent apoptosis (34). These facts have made TLR2 agonists to be attractive adjuvants in the therapy of cancers (28, 30, 35). However, current available TLR2 agonists have limited applications due to their rapid degradation in plasma where abundant esterase exists, which breaking ester bonds connecting glycerol backbone and palmitic acid chains, resulting in diminished stimulation of TLR2. Hence, it is necessary to develop new TLR2 agonists with higher stability *in vivo* and can be further utilized as immune adjuvants.

In this study, we designed a new TLR2 agonist termed SUP3 based on the structure of Pam3CSK4 (Pam3 hereafter), which is a chemically more stable ligand for TLR2/TLR1 heterodimers. Functional analysis revealed that SUP3 had stronger capabilities than Pam3 in DC-mediated activation of CTL and T-dependent antibody production. Further studies of its activity and effects on immune responses showed that SUP3 enhanced antigen presentation ability of cDCs and subsequent T cell activation without leading to excessive inflammatory responses. Our studies demonstrated that SUP3 could function as a potential immune adjuvant in vaccination and immunotherapy.

## Materials and Methods

### Mice

C57BL/6 mice were maintained in a specific pathogen-free facility at Tsinghua University. OT-I and OT-II transgenic mice were purchased from Jackson Laboratory. Mice were sacrificed at the age of 6~8 weeks for experiments. The experiments using mice were approved by the Institutional Animal Care and Use Committees at Tsinghua University.

### Generation of *Tlr2*^−/−^ RAW 264.7 Cells

*Tlr2*^−/−^ RAW 264.7 cells were generated by lentiviral CRISPR-Cas9 system as described previously (36). Briefly, target guide sequences (5′-CACCGCCTGGAGGTTCGCACACGCT-3′, 3′-CGGACCTCCAAGCGTGTGCGACAAA-5′) designed by Genome Engineering 3.0 were ligated into lentiviral vector lentiCRISPR (Addgene 52961). Then lentiCRISPR as well as packaging plasmids pMD2.G (Addgene 12259) and psPax2 (Addgene 12260) were co-transfected into 293FT cells by CaCl_2_ (12.5 mM) for virus packaging. Fresh cell culture medium was changed 12 h post-transfection. After another 72 h, 293FT cell culture supernatant containing virus was harvested, filtered, and concentrated. Then, RAW 264.7 cells were infected by the virus (~5 × 10^5^ TU/mL) with the presence of 8 µg/mL polybrene (Sigma H9268). After that, puromycin (4 µg/mL; Sigma P7255) was added to select positive cells for 48 h post-viral infection. TLR2 depletion efficiency of *Tlr2*^−/−^ RAW 264.7 cells was examined by flow cytometry using a PE conjugated anti-TLR2 antibody (eBioscience, clone 6C2).

### Flow Cytometry

Single cell suspensions were prepared, and cells were stained by fluorescence-conjugated antibodies and viability dye 7-AAD (eBioscience 00-6993) at 4°C for 30 min in the dark. Cells were washed, resuspended, and analyzed on LSR Fortessa (Becton Dickinson). FACS data were analyzed and displayed by FlowJo software (Tree Star).

### Isolation of Splenic DCs and B Cells

Splenic DCs were purified as described previously (37). CD8^+^ cDCs expanded by B16-Flt3L inoculation were enriched, and then CD8^+^ cDCs were positively sorted by MACS technology (Miltenyi Biotec). Splenic B cells were purified through negative selection by Streptavidin-MicroBeads (Miltenyi Biotec 130-048-102) combined with different antibodies cocktail to exclude non-B cells. The antibodies cocktail was composed of biotinylated antibodies against CD4 (T cells and DCs) (eBioscience, clone GK1.5), CD8 (T cells and DCs) (eBioscience, clone 53-6.7), CD43 (T cells, monocytes, granulocytes) (eBioscience, clone eBioR2/60), and Ter119 (erythrocytes) (eBioscience, clone TER119). B cells were further purified through MACS LS column (Miltenyi Biotec 130-042-401).

### Antigen Presentation and Antigen Uptake Assays

Antigen presentation assays were performed as previously described (38). Briefly, ovalbumin (OVA) (10 µg/mL; Sigma A7641) pulsed cDCs were stimulated with or without TLR agonists for 4 h. OT-I CD8^+^ T cells and OT-II CD4^+^ T cells were purified and then labeled by carboxyfluorescein diacetate succinimidyl ester (CFSE) (1 µM; Molecular Probes C34554). CD8^+^ cDCs were cocultured with OT-I CD8^+^ T cells for 60 h, whereas CD11b^+^ cDCs were cocultured with OT-II CD4^+^ T cells for 96 h, both in the presence of granulocyte-macrophage colony-stimulating factor (GM-CSF) (20 ng/mL; Peprotech 315-03). Proliferated OT-I or OT-II T cells were defined as diluted CFSE signal analyzed by flow cytometry. The numbers of T cells were counted by AccuCheck counting beads (Molecular Probes PCB100). Antigen uptake ability of DCs was examined by endocytosis analysis of Alexa Fluor 488 (AF488)-conjugated OVA protein (5 µg/mL; Molecular Probes O-34781) as described previously (39). DCs were incubated with OVA-AF488 (5 µg/mL) at 37°C or on ice (negative control) for 30 min. Cells were washed twice with cold medium and analyzed by flow cytometry.

### TLR Activation and Cytokine Detection

Splenic DCs (5 × 10^5^ cells/mL) were stimulated by Pam3 (200 nM), SUP3 (200 nM), or CpG ODN 1668 (20 nM; Adipogen IAX-200-001) in the GM-CSF (20 ng/mL) supplemented medium for indicated periods. Cell culture supernatants were harvested at indicated time points and stored at −40°C. RAW 264.7 cells and GMDCs were stimulated by lipopolysaccharide (LPS) (20 ng/mL; Adipogen IAX-100-013), Pam3, or SUP3 (both at 200 nM) for 24 h. For *in vivo* administration, Pam3 (2 nmol per mouse), SUP3 (2 nmol per mouse), or LPS (1 µg per mouse) were intravenously (i.v.) injected *via* tail veins. Serum was obtained through tail vein bleeding and centrifuged after coagulation and stored at −40°C. Cytokines (IL-6, eBioscience 88-7064; TNFα, eBioscience 88-7324; IL-12p40, BioLegend 431604), chemokine (MIP1α, R&D Systems DY450-05), and total IgM (eBioscience, 88-50470) were quantified by ELISA kits following the instructions of manufacturers.

### Immunoblot Analysis

Immunoblot assay was performed as described elsewhere (40, 41). Briefly, whole-cell lysates were prepared by direct lysis in SDS loading buffer. Lysates were separated by 10% SDSPAGE and transferred to a polyvinylidene fluoride membrane (Millipore IPVH00010) for probing with antibody. Antibodies against phosphorylated NF-κB p65 (p-p65) (Ser 536) (Clone 93H1), p-SAPK/JNK (Thr183/Tyr185) (Clone 81E11), SAPK/JNK (Clone 56G8), p-p38 (Thr180/Tyr182) (Clone 3D7), and p38 (Clone D13E1) were purchased from Cell Signaling Technology. Antibodies against IκBα (Clone 93H1), p-ERK (Clone E-4), and ERK (Clone K-23) were purchased from Santa Cruz Biotech. The chemiluminescence signals were captured by Amersham Image 600 system (GE Healthcare).

### OVA-Coated Splenocytes (OCS) Immunization

Splenocytes were X-ray irradiated (1500 Rad) and then coated with OVA (1 mg per spleen) in RPMI 1640 medium (Gibco 11875-119) for 30 min at 37°C. Cells were washed twice with RPMI 1640 medium. Then OCS (2 × 10^7^ cells per mouse) were i.v. injected through tail vein, together with or without Pam3 (2 nmol per mouse) or SUP3 (2 nmol per mouse). Seven days post-immunization, OVA-specific CD8^+^ T cells in spleen were analyzed by flow cytometry by staining with fluorescence-conjugated antibodies against CD19 (eBioscinece, clone 1D3), CD3 (eBioscinece, clone 145-2C11), CD8 (eBioscinece, clone 53-6.7), and SIINFEKL/H-2Kb-Pentamer (ProImmune F093-4A).

### Tumor Model

Tumor model was applied as previously described with some modifications (42). Briefly, mice were immunized by OCS alone, OCS with Pam3 (2 nmol per mouse), or OCS with SUP3 (2 nmol per mouse) for 30 days. Then mice were i.v. injected with OVA-expressing B16 melanoma cells (B16-OVA) (5 × 10^5^ cells per mouse). After 16 days, the numbers of metastatic tumors in lung were counted.

### OT-II Activation *In Vivo*

Carboxyfluorescein diacetate succinimidyl ester-stained OT-II CD4^+^ T cells (2 × 10^6^ cells per mouse) were transferred into mice 1 day prior immunization. Then the recipients were subcutaneously (s.c.) injected with OVA alone (100 µg per mouse), or OVA mixed with SUP3 (2 nmol per mouse) or Pam3 (2 nmol per mouse) at groin. Three days after immunization, OT-II CD4^+^ T cells from inguinal lymph nodes were analyzed by flow cytometry.

### Thymus-Dependent (TD) Antigen Immunization and Antibody Titer Analysis

Thymus-dependent antigen immunization and antibody titer analysis were performed as previously described (43). Briefly, wild-type (WT) mice were immunized by i.p. injection of TD antigen nitrophenyl-keyhole limpet hemocyanin (NP-KLH, 5 µg per mouse; Biosearch Technologies N-5060) alone, or mixed with SUP3 (2 nmol per mouse), Pam3 (2 nmol per mouse), or alum (3 µg per mouse; Thermo 77161). Serum was harvested every week, and nitrophenyl-specific IgM and IgG were determined by ELISA. IgM and total IgG were captured by nitrophenyl 30mer (Biosearch Technologies N-5050H), and high affinity IgG was captured by nitrophenyl 8mer (Biosearch Technologies N-5050L) pre-coated ELISA plates (Corning 9018).

### Antigen Deposition

Wild-type mice were s.c. injected with OVA-AF647 alone (25 µg per mouse; Molecular Probes O-34784), or mixtures of OVA-AF647 with SUP3 (2 nmol per mouse) or Pam3 (2 nmol per mouse) at groin. Inguinal lymph nodes were isolated 24 h post-injection, and then immune cells were analyzed by flow cytometry.

### Statistical Analysis

Data were analyzed by ANOVA followed by Bonferroni multiple comparison test or by unpaired, two-tailed Student’s *t* test with GraphPad Prism 5. Data were presented as mean ± SD or ± SEM, and *p* < 0.05 (*) was considered statistically significant.

## Results

### Design of TLR2 Agonist SUP3

**Figure 1:**
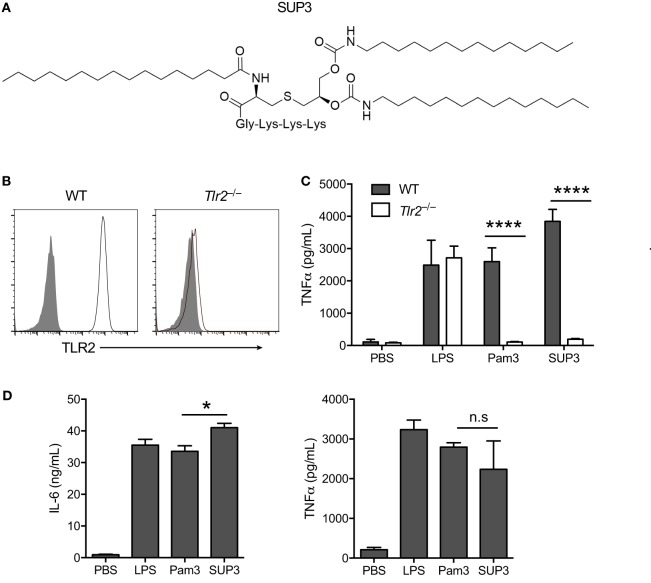
**Specificity and activity of novel TLR2 agonist SUP3**. **(A)** Schematic chemical structure of SUP3. **(B)** Surface expression of TLR2 in wild-type (WT) and *Tlr2*^−/−^ RAW 264.7 cell lines. Gray shadows represented isotype control and black lines represented TLR2. **(C)** TNFα production by RAW 264.7 cells upon stimulation with lipopolysaccharide (LPS) (1 µg/mL), Pam3CSK4 (Pam3 hereafter) (500 nM), or SUP3 (500 nM) for 8 h. **(D)** GMDCs were generated by culturing bone marrow cells with granulocyte-macrophage colony-stimulating factor and interleukin-4 for 7 days, then activated by LPS (20 ng/mL), Pam3 (200 nM), or SUP3 (200 nM) for 24 h. The production of cytokines IL-6 and TNFα in culture supernatant was determined by ELISA. *p < 0.05, ****p < 0.0001, “n.s.” indicates statistically non-significant.

Pam3 is a lipopeptide and serves as a TLR2 agonist through binding to TLR2/TLR1 heterodimer. Although Pam3 is widely used as a TLR2 agonist, some intrinsic problems remain in detailed mechanism studies and drug development. The two ester groups of Pam3 can be easily hydrolyzed in plasma, and the hydroxyl group in serine could be readily oxidized in physiological conditions (Figure S1A in Supplementary Material). All of these facts made Pam3 less stable *in vivo*. In order to overcome the disadvantages of the current existing TLR2 ligands, we designed a new TLR2 agonist that should be chemically and metabolically more stable. Based on the structural features of Pam3 and TLR1/2 receptors co-crystal, we replaced the two ester-linked palmitoyl groups with chemically more stable bioisostere, carbamate-linked C14 groups (Figures S1A,B in Supplementary Material). In addition, we displaced serine residue in Pam3 with glycine residue to eliminate this chiral center (Figures S1A,B in Supplementary Material). Since the third lysine residue in Pam3 is crucial for H bonding, we kept three lysine residues in the new molecule and removed the forth lysine residue to further reduce the molecular weight of the new TLR2 agonist, (2S,5S,8S,14R,18R)-2,5,8-tris(4-aminobutyl)-4,7,10,13,21-pentaoxo-14-palmitamido-18-((tetradecylcarbamoyl)oxy)-20-oxa-16-thia-3,6,9,12,22-pentaazahexatriacontan-1-oic acid. It is termed SUP3 (Figure 1A; Figures S1A,B in Supplementary Material). Chemical characterization of SUP3 by ^1^H nuclear magnetic resonance and electrospray ionization mass spectrum analysis further confirm the structure (Figures S2A,B in Supplementary Material). The affinity of SUP3 is comparable with Pam3 (Figure S3A in Supplementary Material), whereas the plasma stability of SUP3 is much higher than Pam3 (Figure S3B in Supplementary Material).

To validate the specificity of SUP3 for TLR2, we generated *Tlr2*^−/−^ RAW 264.7 cells with CRISPR-Cas9 system (Figure 1B). Both Pam3 and SUP3 stimulation induced large amount of TNFα production by WT RAW cells, indicating that SUP3 could function similarly as Pam3 in triggering TLR2 response (Figure 1C). TLR2 deficiency abolished the effect of Pam3 and SUP3 on RAW cells, evidenced by no secretion of TNFα by *Tlr2*^−/−^ RAW cells upon challenge by Pam3 and SUP3 (Figure 1C). It demonstrated that TLR2 was the receptor for SUP3. To further determine the bioactivity of SUP3 as a TLR2 agonist, we determined the cytokine production by GMDCs upon TLR2 stimulation. GMDCs were generated from bone marrow cells cultured in the presence of GM-CSF and interleukin-4 (44, 45). It has been demonstrated that GMDCs are potent pro-inflammatory cytokine producers upon TLR stimulation and GMDCs express TLR2 and respond to TLR2 ligands. We found that SUP3 exhibit equal ability as Pam3 in activating GMDCs, as the production of IL-6 and TNFα were comparable by GMDCs upon stimulation by SUP3 and Pam3 (Figure 1D). Taken together, these data suggested that SUP3, the newly designed TLR agonist, engaged TLR2 specifically as well as Pam3.

### SUP3 Induced More Vigorous CTL Responses Than Pam3 without Excessive Production of Inflammatory Cytokines *In Vivo*

**Figure 2:**
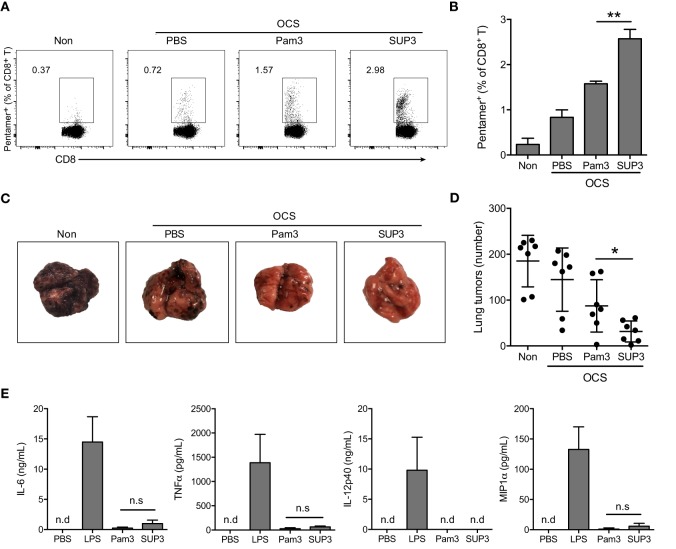
**SUP3 induced more vigorous cytotoxic T lymphocytes (CTLs) responses than Pam3CSK4 without excessive production of inflammatory cytokines *in vivo***. **(A,B)** Mice were immunized by i.v. injection of ovalbumin (OVA)-coated splenocytes (OCS) (2 × 10^7^ cells per mouse) alone, OCS with SUP3 (2 nmol per mouse) or Pam3 (2 nmol per mouse) for 7 days. The percentages of pentamer positive cells (CD19^−^ CD3^+^ CD8^+^ SIINFEKL/H-2Kb-Pentamer^+^) in spleens, i.e., the OVA-specific CD8^+^ T cells, were determined by flow cytometry **(A)** and the statistic calculation is presented in **(B)**. Representative data from three independent experiments with three mice in each group were presented. **(C,D)** Mice were immunized as in **(A)** for 30 days, then inoculated with OVA-expressing B16 melanoma cells (B16-OVA) (5 × 10^5^ cells per mouse) by i.v. injection and analyzed for metastatic tumor formation in the lung 16 days after inoculation. Data from two independent experiments with three mice in each group were shown. Representative tumor formations in the lung from each group were displayed in **(C)**, and the number of metastatic tumors was calculated in **(D)**. **(E)** Mice were i.v. injected with SUP3 (2 nmol per mouse), Pam3 (2 nmol per mouse), or lipopolysaccharide (LPS) (1 µg per mouse) for 1 h, the serum was collected, and the levels of different cytokines were determined by ELISA. “n.d.” indicates “not detected.” *p < 0.05, **p < 0.01.

The consequence of immunization or vaccination is the acquirement of preventive or therapeutic effects on infections or tumors, *via* generating antigen-specific adaptive immune responses and memory. Elimination of tumors or intracellular infection requires antigen-specific CD8^+^ CTLs, the major executors in cellular immunity (46, 47). In mice, the differentiation and activation of CD8^+^ CTL is launched through antigen cross-presentation by CD8^+^ cDC (46, 47). Some TLR agonists have been shown to enhance antigen cross-presentation by cDCs and the subsequent CD8^+^ T cell activation (30, 35, 48–51). To investigate whether SUP3 has an effect on CTL responses, we determined the activity of SUP3 in antigen-specific cellular immunity. Mice were immunized by intravenous injection of OVA coated splenocytes (OCS), as these cell-associated OVA antigens can be cross-presented efficiently by CD8^+^ cDC to CD8^+^ T cells (52). Seven days post-immunization, the percentages of OVA antigen-specific CD8^+^ T cells in spleens were determined by pentamer (SIINFEKL/H-2Kb) staining. The effects of various doses of SUP3 and Pam3 on the induction of CTL were determined and 2 nmol was selected as the optimal dose in the following assays (Figures S4A,B in Supplementary Material). Administration of SUP3 at a high dose of 10 nmol resulted in decreased CTL induction and enlarged spleens, whereas treatment with Pam3 at the same dose did not have the same effect (data now shown), indicating that SUP3 was much more potent than Pam3 and the mice could not tolerate such a high dose of agonists. We found that OCS formulated with SUP3 induced significantly more antigen-specific CD8^+^ T cells than that with Pam3 *in vivo* (Figures 2A,B). To further determine whether the enhanced CTL activation by SUP3 can lead to more vigorous antitumor activity, we immunized mice as described above and then challenged by intravenous inoculation of OVA-expressing B16 melanoma cells (B16-OVA) (53). We found that mice immunized with OCS and SUP3 showed markedly reduced number of tumors in the lungs compared to those with OCS alone or OCS with Pam3 16 days after the challenge (Figures 2C,D). It indicated that immunization with SUP3 conferred more efficient CTL responses, thus more effective protection against tumor formation. Taken together, these results demonstrated that SUP3 was far more effective than Pam3 in inducing CTL responses *in vivo*.

Sustainable stimulation by TLR agonists such as LPS, often lead to elevated inflammatory cytokines *in vivo*, which may result in severe adverse reactions. To test whether this is also the case for SUP3 and Pam3, we determined the level of serum inflammatory cytokines upon SUP3 or Pam3 administration. The inflammatory cytokines including IL-6, TNFα, and IL-12p40 and chemokine MIP1α were all maintained at low levels even after SUP3 or Pam3 stimulation (Figure 2E), suggesting that both SUP3 and Pam3 acted as mild TLR agonists for inflammatory responses. Overall, SUP3 promoted stronger CTL response without inducing excessive inflammatory cytokine production, suggesting that SUP3 could function as a better adjuvant than Pam3 with potential for clinical applications.

### SUP3 Enhanced Cross-Presentation of OVA Antigen by CD8^+^ cDCs *In Vitro*

**Figure 3:**
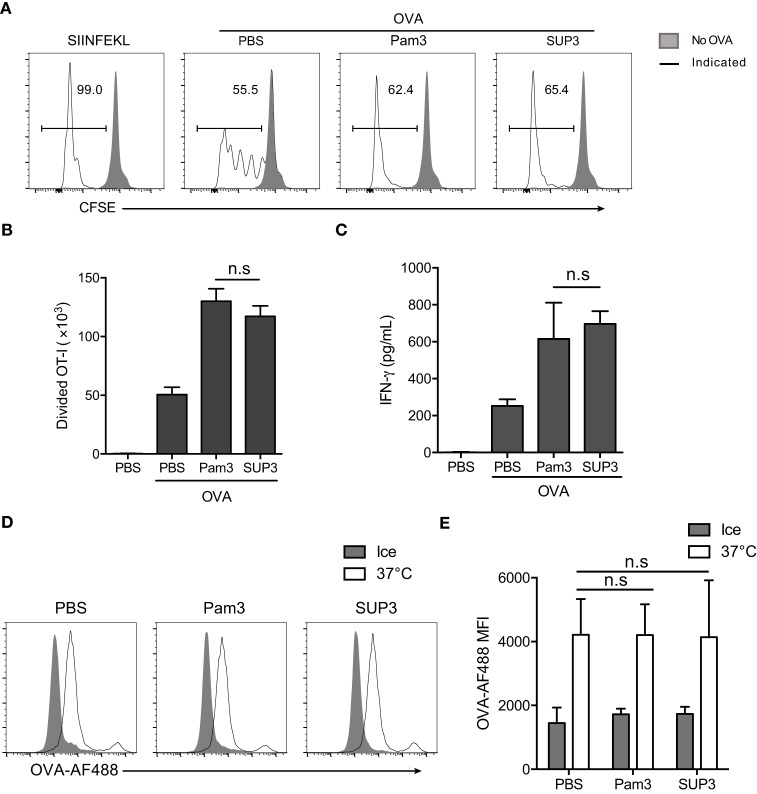
**SUP3 facilitated cross-presentation of ovalbumin (OVA) antigen by CD8^+^ conventional dendritic cells (cDCs) *in vitro***. **(A–C)** Purified splenic CD8^+^ cDCs were stimulated with or without Pam3 (200 nM) or SUP3 (200 nM) for 4 h, then pulsed by OVA protein (20 µg/mL) for another 4 h. Cells were washed and cocultured with carboxyfluorescein diacetate succinimidyl ester (CFSE)-labeled OT-I CD8^+^ T cells for 60 h and analyzed by flow cytometry. **(A)** The dividing OT-I cells were measured by reduced level of CFSE-labeling. Representative data from three independent experiments with triplicated samples for each group was shown. “No OVA” (gray shadow) represented negative control that untreated DCs cocultured with OT-I. “Indicated” (solid line) represented DCs pulsed by peptide SIINFEKL (the first), or OVA proteins (the rest) which were also pretreated by PBS, Pam3, or SUP3, respectively. **(B)** Absolute numbers of proliferated OT-I T cells were calculated. **(C)** Interferon-γ from the supernatant of coculture was determined by ELISA 24 h later. Pooled data of three independent experiments with triplicated samples for each group are shown. **(D,E)** Purified splenic CD8^+^ cDCs were pretreated by Pam3 (200 nM), SUP3 (200 nM), or PBS for 4 h, then incubated with OVA-Alexa Fluor (AF) 488 (5 µg/mL) at 37°C or on ice (negative control) for 30 min. The cells were then washed with cold PBS and analyzed by flow cytometry **(D)**. The mean fluorescence intensities (MFI) of AF488 were shown in **(E)**.

Cytotoxic T lymphocyte is one of the major effector cell types in cellular immunity, which can be induced by CD8^+^ cDCs *via* antigen cross-presentation to naïve CD8^+^ T cells (11, 13, 14, 47). It is well known that TLR activation can enhance the function of DCs (54). It is likely that SUP3 promoted stronger CTL responses through enhancing the function of CD8^+^ cDCs. To investigate whether SUP3 played any roles in antigen cross-presentation by splenic CD8^+^ cDCs and the subsequent activation of naïve CD8^+^ T cells, we examined the antigen cross-presentation capacity of CD8^+^ cDCs with or without SUP3 by using OVA protein antigen and the OVA-specific T cell receptor (TCR) transgenic OT-I CD8^+^ T cells. The CD8^+^ cDCs pulsed with OVA peptide SIINFEKL specifically recognized by OT-I T cells were used as positive control. SIINFEKL peptide pulsed CD8^+^ cDCs presented antigen peptide directly without antigen processing and induced strong OT-I T cell proliferation (Figure 3A). Upon stimulation of OVA protein pulsed CD8^+^ cDCs by TLR2 agonists, the proliferation of cocultured OT-I cells increased significantly, evidenced by increased numbers of divided OT-I T cells (Figures 3A,B) and enhanced production of IFN-γ (Figure 3C) compared to that without stimulation by TLR2 agonists. These results indicated that SUP3 and Pam3 were comparable in enhancing cross-presentation by CD8^+^ cDCs *in vitro*, thus leading to enhanced antigen-specific CD8^+^ T cell activation. It is interesting to note that unlike the results described above, in which SUP3 induced much stronger antigen-specific CD8^+^ T cell proliferation and antitumor CTL responses *in vivo*, SUP3 and Pam3 induced similar levels of OVA-specific CD8^+^ T cell activation *in vitro*. This difference is likely due to the improved stability of SUP3 compared to Pam3 *in vivo*.

Exogenous antigens are internalized, degraded into short peptides, uploaded onto MHC-I and finally transported to cell surface for recognition by cognate T cells (55–57). The first step of antigen presentation is internalization of extracellular materials. To clarify whether antigen uptake process is affected by TLR2 engagement, we determined the endocytosis of fluorescence-conjugated soluble OVA (OVA-Alexa Fluor 488, OVA-AF488) by CD8^+^ cDCs. The results showed that the uptake of OVA by CD8^+^ cDCs was not influenced by TLR2 activation (Figures 3D,E), indicating that enhanced antigen presentation by CD8^+^ cDCs was not due to facilitated antigen uptake process.

### SUP3 Upregulated Expression of Costimulatory Molecules on CD8^+^ cDCs and Induced Low Level Production of Inflammatory Cytokines

**Figure 4:**
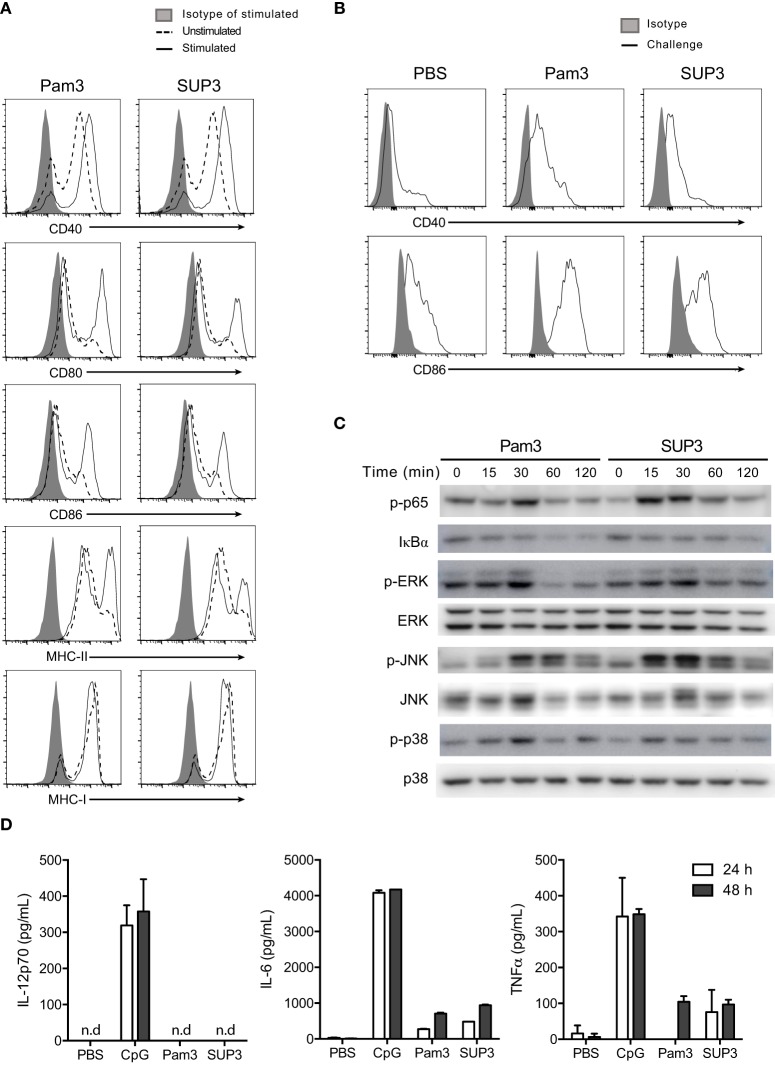
**SUP3 stimulated maturation of CD8^+^ conventional dendritic cells (cDCs)**. **(A)** Purified CD8^+^ cDCs were treated with SUP3 (200 nM) or Pam3 (200 nM) in the presence of granulocyte-macrophage colony-stimulating factor (GM-CSF) (20 ng/mL) for 24 h. The surface expression of costimulatory and major histocompatibility complex (MHC) molecules were then analyzed by flow cytometry. Representative data from three independent experiments were shown. “Isotype” represented relative isotype antibody staining of Pam3 or SUP3 stimulated cells. **(B)** SUP3 (2 nmol per mouse), Pam3 (2 nmol per mouse), or PBS were i.v. injected into mice for 1 h. Splenic CD8^+^ cDCs were enriched, and surface expression of CD40 and CD86 was analyzed by flow cytometry. Representative data from two independent experiments with three mice for each group were shown. **(C)** Purified CD8^+^ cDCs were treated with SUP3 (200 nM) or Pam3 (200 nM) for indicated periods (0~120 min), and western blot analysis was performed with the cell lysate for phosphorylated p65 (p-p65), IκBα, p-ERK, ERK, p-JNK, JNK, p-p38, and p38. Blot of p38 was also regarded as loading control. **(D)** Purified CD8^+^ cDCs were treated with CpG 1668 (20 nM), SUP3 (200 nM), or Pam3 (200 nM) in the presence of GM-CSF (20 ng/mL). Supernatants were harvested 24 and 48 h post-stimulation, and cytokines were measured by ELISA. Representative data from two independent experiments with triplicated samples for each group were shown.

Costimulatory molecules, which serve as the second signal for T cell activation, can be efficiently upregulated upon TLR activation. To investigate whether SUP3 affected the expression of costimulatory molecules on CD8^+^ cDCs, we stimulated purified DCs by SUP3 or Pam3 *in vitro* and stained with antibodies to costimulatory molecules as well as MHC molecules. We found that both SUP3 and Pam3 induced substantial upregulation of CD40, CD80, CD86, and MHC-II on CD8^+^ cDCs, but not MHC-I (Figure 4A), demonstrating that SUP3 induced effective costimulation as well as Pam3 *in vitro*. We then tested whether SUP3 had similar effects on splenic CD8^+^ cDCs *in vivo*. Upon *in vivo* administration of SUP3 or Pam3 *via* intravenous (i.v.) route, the expressions of CD40 and CD86 increased on splenic CD8^+^ cDCs from both SUP3- or Pam3-treated mice, indicating the ability of SUP3 to promote DC activation *in vivo* (Figure 4B).

Toll-like receptor stimulation is required for functional maturation of DCs, which is essential for T cell activation and initiation of adaptive immunity. Antigen recognition without danger signals like TLR ligands leads to tolerance but not antigen processing for subsequent presentation (17, 58). It is well established that TLR-induced activation of nuclear factor-kappa B (NF-κB) and mitogen-activated protein kinase (MAPK) signaling events contribute to activation of responding cells. To determine whether SUP3 could activate classical Toll-dependent signaling pathways in CD8^+^ cDCs, we treated purified CD8^+^ DCs by TLR2 agonists at various time points (0–120 min) and detected the expression of the relevant signaling molecules and their phosphorylated forms by immunoblotting. We found SUP3 stimulation induced phosphorylation of p65 and degradation of IκBα and enhanced phosphorylation of ERK, JNK, and p38 in CD8^+^ cDCs (Figure 4C), indicating that similar to Pam3, SUP3 was able to induce activation of NF-κB and MAPK pathways. Taken together, SUP3 could induce activation of canonical TLR signaling events in CD8^+^ cDCs.

In addition to costimulation, secretion of cytokines by DCs is another major feature of DC activation upon TLR engagement. Cytokines like bioactive IL-12p70 predominately produced by CD8^+^ cDCs drive functional differentiation of activated T cells and provide the third signal for DC-induced T cell activation (59). As SUP3 could facilitate CD8^+^ T cell activation (Figures 3A–C), we therefore examined whether SUP3 could induce inflammatory cytokines production by CD8^+^ cDCs. We activated purified CD8^+^ cDCs by SUP3, Pam3, or CpG *in vitro* and quantified the amounts of cytokines in the supernatant by ELISA. Interestingly, in contrast to CpG stimulation which induced large amount of IL-12p70, IL-6, and TNFα production by CD8^+^ cDCs, SUP3 and Pam3 induced no IL-12p70 and only low levels of IL-6 and TNFα (Figure 4D). These results demonstrated that SUP3 acted as a mild agonist in inflammatory responses. Collectively, our results demonstrated that SUP3 acted as an efficient enhancer of antigen cross-presentation by CD8^+^ cDCs through upregulating surface costimulatory molecules and promoting activation of NF-κB and MAPK signaling pathways, without inducing large amount of inflammatory cytokines. Thus, SUP3 fulfilled the properties of a good adjuvant with strong immunostimulatory capacity for T cell activation and minimal induction of inflammatory response.

### SUP3 Enhanced CD4^+^ T Cell Activation and Production of Antibodies against TD Antigens

**Figure 5:**
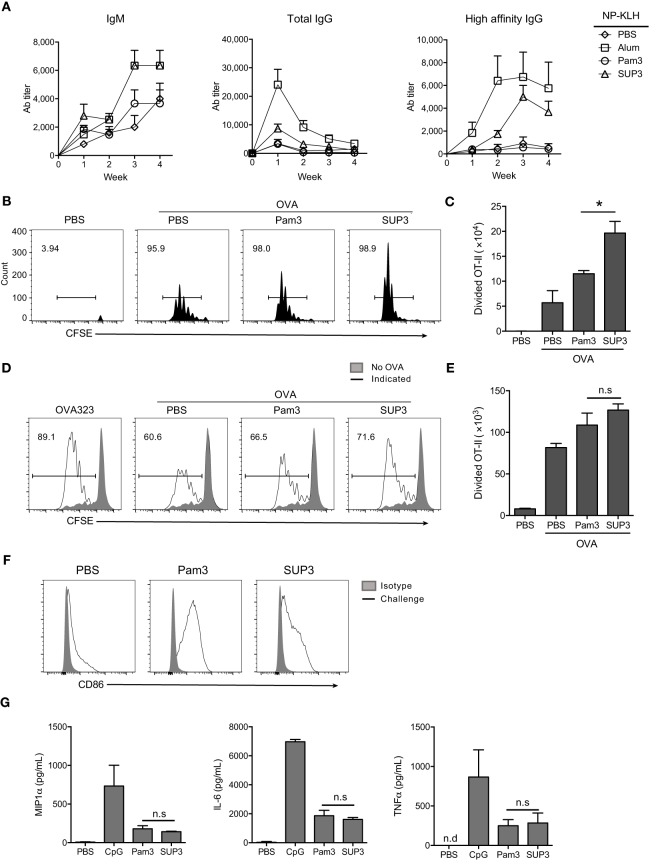
**SUP3 enhanced antigen presentation ability of CD11b^+^ conventional dendritic cells (cDCs) and production of antibodies against thymus-dependent (TD) antigens**. **(A)** Mice were immunized by TD antigen nitrophenyl-keyhole limpet hemocyanin (NP-KLH) (5 µg per mouse) with or without SUP3 (2 nmol per mouse), Pam3 (2 nmol per mouse), or alum (3 µg per mouse) as adjuvants, respectively. Serum IgM (nitrophenyl 30mer-captured, NP30-captured), total IgG (NP30-captured), and high affinity IgG (nitrophenyl 8mer-captured, NP8-captured) specific to NP at indicated time points were examined by ELISA. **(B,C)** Carboxyfluorescein diacetate succinimidyl ester-labeled OT-II CD4^+^ T cells (2 × 10^6^ cells per mouse) were transferred into mice 1 day prior immunization. Then the recipients were subcutaneously (s.c.) injected with ovalbumin (OVA) alone (100 µg per mouse), or OVA (100 µg per mouse) mixed with SUP3 (2 nmol per mouse) or Pam3 (2 nmol per mouse) at groin. Three days after immunization, division of OT-II CD4^+^ T cells from inguinal lymph nodes were analyzed by flow cytometry **(B)**. Absolute numbers of proliferated OT-II CD4^+^ T cells were calculated in **(C)**. Pooled data were from two independent experiments with three mice in each group. “No OVA” (gray shadow) represented negative control that untreated DCs cocultured with CSFE-labeled OT-II. “Indicated” (solid line) represented DCs pulsed by peptide OVA323 (the first), or OVA proteins (the rest) which were also pretreated by PBS, Pam3, or SUP3, respectively. **(D,E)** Purified splenic CD11b^+^ cDCs were stimulated by Pam3 (200 nM) or SUP3 (200 nM) for 4 h then pulsed by OVA (20 µg/mL) for another 4 h. Cells were then thoroughly washed and cocultured with CFSE-stained OT-II CD4^+^ T cells for 4 days and analyzed by flow cytometry. **(D)** The division of OT-II cells was measured by reduced level of CFSE-labeling. **(E)** Absolute numbers of proliferated T cells from **(D)** were calculated. **(F)** SUP3 (2 nmol per mouse), Pam3 (2 nmol per mouse), or PBS were i.v. injected into mice for 1 h, and splenic CD11b^+^ cDCs were enriched and analyzed for CD86 expression by flow cytometry. “Isotype” represented relative isotype antibody staining of Pam3 or SUP3 stimulated cells. **(G)** Purified CD11b^+^ cDCs were activated by SUP3 (200 nM), Pam3 (200 nM), or CpG (20 nM) in granulocyte-macrophage colony-stimulating factor (20 ng/mL) containing medium for 24 h. The cytokines from supernatant were determined by ELISA (*p < 0.05, **p < 0.01).

In addition to cellular immunity, the other arm of adaptive immunity is humoral immunity mediated by antibodies. Production of TD antigen-specific antibodies requires the assistance of Th2 subtypes, which is mainly induced by CD11b^+^ cDCs (15, 60, 61). Exogenous antigens are routinely presented *via* MHC-II pathway by CD11b^+^ cDCs and ultimately recognized by CD4^+^ T cells for helper T cell differentiation (11). CD11b^+^ cDCs expressed comparable levels of TLR2 to that of CD8^+^ cDCs (Figure S5A in Supplementary Material). We predicted that SUP3 might enhance the functions of CD11b^+^ cDCs thus Th2-mediated production of TD antigen-specific antibodies. To test this hypothesis, we immunized mice *via* intraperitoneal (i.p.) injection of TD antigen NP-KLH with or without TLR agonists. Formulation with alum was regarded as positive control in this setting, as alum is always used as an efficient adjuvant in antibody induction. Serum antibody titers were determined every week post-immunization. As expected, SUP3 induced higher titers of TD antigen-specific antibodies including IgM, total IgG and high affinity IgG than Pam3 did (Figure 5A). This result suggested that SUP3 remained functioning for a longer time than Pam3 *in vivo*, thus resulting in higher levels of antibody production.

As mentioned above, CD4^+^ Th2 cells polarized by CD11b^+^ cDCs are critical in TD antigen-specific antibodies production. Thus, SUP3 might enhance TD antibody production *via* facilitating CD4^+^ T cell activation *in vivo*. To test this hypothesis, purified CD4^+^ OT-II T cells from spleens of OVA-specific TCR transgenic mice were labeled by CFSE and transferred into WT mice for 1 day, followed by subcutaneous (s.c.) immunization with soluble OVA proteins with TLR agonists for another 3 days. Treatment with SUP3 induced more proliferation of OT-II CD4^+^ T cells than that with Pam3 in draining lymph nodes (Figures 5B,C), demonstrating the ability of SUP3 to induce stronger CD4^+^ T cell activation *in vivo*, thus leading to enhanced TD antibodies production.

### SUP3 Facilitated Antigen Presentation by CD11b^+^ cDCs *In Vitro*

As CD4^+^ T cell activation is enhanced upon SUP3 treatment, we speculated that SUP3 functioned through improving functions of CD11b^+^ cDCs. To test this speculation, we evaluated OVA antigen presentation by SUP3 pretreated CD11b^+^ cDCs to OT-II CD4^+^ T cells. An enhanced OT-II CD4^+^ T cell proliferation was observed upon stimulation of CD11b^+^ cDCs by SUP3 *in vitro* (Figures 5D,E), indicating that SUP3 strengthened antigen presentation ability of CD11b^+^ cDCs. The maturation of CD11b^+^ cDC is marked by upregulation of costimulatory molecules. Intravenous administration of SUP3 and Pam3, both induced maturation of splenic CD11b^+^ cDC indicated by CD86 upregulation (Figure 5F). Furthermore, similar to CD8^+^ cDCs, CD11b^+^ cDCs produced lower level of IL-6, TNFα, and MIP1α upon stimulation by SUP3 and Pam3, implying a weak ability of SUP3 in inducing inflammatory responses (Figure 5G).

Collectively, these results demonstrated that SUP3 facilitated antigen presentation by CD11b^+^ cDCs and subsequent activation of OT-II CD4^+^ T cells, which led to higher levels of TD antibody production *in vivo*. These observations again demonstrated the physiological advantages of SUP3 over Pam3 as an immune adjuvant.

### SUP3 Enhanced Antigen Deposition in APCs in Draining Lymph Nodes

**Figure 6:**
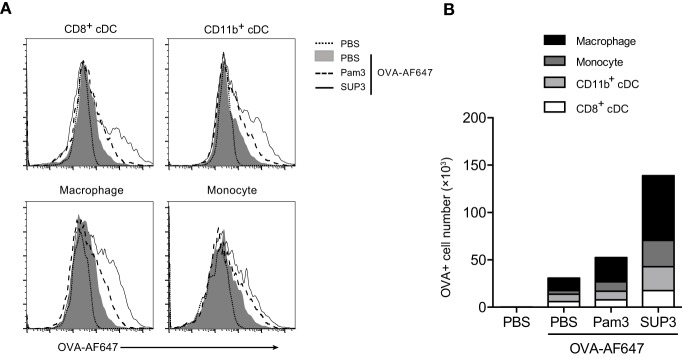
**SUP3 enhanced antigen deposition in draining lymph nodes**. Mice were immunized at groin by s.c. injection of ovalbumin (OVA)-Alexa Fluor 647 (AF647) (25 µg per mouse) with or without SUP3 (2 nmol per mouse) or Pam3 (2 nmol per mouse) for 24 h. Inguinal lymph nodes were isolated, and immune cells were analyzed for levels of AF647 fluorescence by flow cytometry **(A)**. The number of OVA-AF647 positive cells was calculated in **(B)**. Representative data were from two independent experiments with three mice in each group.

As immune adjuvants enhance immunogenicity of simultaneously administrated antigens *via* increasing antigen deposition in lymph nodes, and prolonging antigen presentation (62, 63), we proposed that SUP3 might prolong the duration of antigen deposition in draining lymph nodes. To test this, we s.c. immunized mice with OVA-AF647 at groin, or co-administrated with either SUP3 or Pam3 for 24 h. Antigen deposition in various immune cell populations from inguinal lymph nodes was then assessed. After immunization of OVA with SUP3, increased numbers of antigen-containing CD8^+^ cDCs, CD11b^+^ cDCs, macrophages, and monocytes were observed compared to those with OVA alone or OVA with Pam3 (Figures 6A,B). Moreover, the total number of cells containing antigens increased more upon administration of SUP3 over Pam3 (Figure 6B). This finding indicated that SUP3 facilitated antigen deposition in cDCs, macrophages and monocytes in draining lymph nodes, thus contributed to enhanced T cell activation.

### SUP3 Directly Promoted Stronger B Cell Activation *In Vitro*

**Figure 7:**
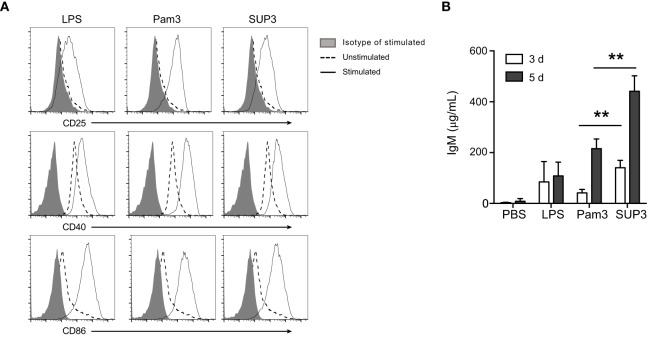
**SUP3 promoted stronger B cell activation *in vitro***. Purified splenic B cells (2 × 10^6^ cells/mL) were treated with SUP3 (200 nM), Pam3 (200 nM), or lipopolysaccharide (LPS) (1.5 µg/mL), respectively. **(A)** Surface CD25, CD40, and CD86 were stained 24 h post-stimulation. **(B)** Supernatants were harvested 3 or 5 days post-stimulation, and total IgM in the supernatant was determined by ELISA. Representative data was from two independent experiments with triplicated wells in each group (**p < 0.01).

B lymphocytes can function as APCs that directly respond to invading pathogens through recognition *via* innate sensors. In addition to myeloid cells including DCs, macrophages, and monocytes, B cells are only lymphocytes positive for TLR2 (Figure S5A in Supplementary Material). TLR ligation on B cells drives fast IgM production to eliminate pathogens before the formation of effective antibody-mediated adaptive immunity, which requires 5–7 days to develop (64). The direct TLR triggered IgM production by B cells, as a thymus independent (TI) pathway, compensates the delayed formation of humoral immunity to control pathogens. To evaluate B cell response to SUP3, we analyzed the expression of activation markers and production of IgM upon treatment by SUP3 *in vitro*. Engagement of TLR2 by SUP3 and Pam3 induced marked upregulation of CD25, CD40, and CD86 on B lymphocytes (Figure 7A), implying a direct effect of SUP3 on B cell activation. In addition, more antigen-independent production of IgM was induced by SUP3 than that induced by Pam3 (Figure 7B), indicating that SUP3 could induce a stronger and longer lasting B cell response than Pam3. This finding is in line with the better stability of SUP3 compared to Pam3. Our results demonstrated that TLR2 agonists could enhance B cell function not only in T cell-dependent but also in T-independent manners.

## Discussion

Our newly designed TLR2 agonist SUP3 is structurally similar to classical TLR2 ligand Pam3. The chemical modification by replacing oxygen with nitrogen in Pam3 stabilizes the covalent bonds between glycerol backbone and palmitic acid chains. In addition, glycine instead of lysine reduces the chiral center and further simplifies the structure. These improvements overcome current disadvantages of Pam3, such as weak stability and structural complexity. Meanwhile, the specificity and affinity to TLR2 and bioactivity to promote TLR2-dependent cytokines are retained. Moreover, the peptide components of SUP3 make it easy and convenient to be conjugated with antigenic peptides. This type of conjugation could easily confer adjuvant activity to the conjugated antigens and induce markedly enhanced immune responses toward antigens without additional adjuvant formulations (6, 25, 65, 66). Intriguingly, this special feature is not shared with any other TLR agonists except those of TLR2. Adjuvants of vaccines are defined as components capable of enhancing and shaping antigen-specific immune responses (1). Ideal adjuvants should possess properties including safety and effectiveness with defined and well-controlled functions (1). An effective adjuvant should help to reduce required dose of antigens or the number of immunizations and have significant improvement in antibody- as well as cell-mediated immune responses (1). Although both SUP3 and Pam3 possess these features of an ideal adjuvant, SUP3 is chemically more stable and structurally simpler than Pam3. SUP3 also exhibited clear advantages compared to Pam3 in biological activities, suggesting that SUP3 could be a good substitution of Pam3 in vaccinations and cancer treatment.

Recognition of microbial or viral components by PRRs on DCs elicits immediate host response and ultimately long-lasting adaptive immunity (17). DCs are one of the first encounters for non-self components, such as pathogens and tumor antigens. The initial responses of DCs to these pathogens determine the shape of adaptive immunity. Thus DCs occupy an indispensable position in orchestrating immune responses to eliminate pathogens and tumors. Consequently, immune modulations targeting DCs could be an effective strategy to harness immune system to endow protective immunity. Cancer immunotherapy with DCs is favorable due to fewer side effects, although with limited benefit observed from recent studies (67).

Enhancement of adaptive immunity is a key parameter of adjuvants in the context of vaccines. Shaping adaptive immunity always requires several days of time. In this study, we observed that more antigen-specific CTLs were generated in the presence of SUP3 than that with Pam3 during the induction of effector T cell differentiation by OCS *in vivo*. Consistent with this observation, the presence of SUP3 induced much stronger protective response against tumor challenge, as revealed by significantly reduced tumor formation in the lungs. Those effects of SUP3 could be achieved through enhancing antigen cross-presentation by CD8^+^ cDCs, which are responsible for CTL induction and subsequent clearance of tumor cells. SUP3 significantly enhanced the surface expression of costimulatory molecules on cDCs, providing one of the essential signals for T cell activation. Intriguingly, SUP3 enhanced antigen cross-presentation by CD8^+^ cDCs was accompanied by a low to moderate levels of inflammatory cytokines, which is different from effects of many other TLR agonists used as adjuvant such as LPS. This functional feature of SUP3 can be considered as an advantage for a good adjuvant, as over production of inflammatory cytokines could be detrimental. Altogether, SUP3 can serve as a potential adjuvant in therapeutic or prophylactic vaccines.

Antigen-specific antibody-mediated humoral immunity is the other arm of adaptive immunity. Indeed, SUP3 not only enhances CTL-mediated cellular immunity but also facilitates the production of various types of specific antibodies to TD antigens. As mentioned previously, TD antibody production requires effector CD4^+^ T cells activated through MHC-II antigen presentation pathway mainly promoted by CD11b^+^ cDCs. Addition of SUP3 enhanced antigen presentation ability of CD11b^+^ cDCs in *in vitro* assays and administration of SUP3 enhanced CD4^+^ T cell activation *in vivo*. Thus, SUP3 induced more antibody production than Pam3 in T-dependent manner through enhancing antigen presentation by CD11b^+^ cDCs. Moreover, an enhanced TI antibody production by B cells was also observed after SUP3 administration. B cells are capable of recognizing and responding to pathogens directly *via* innate immune receptors like TLR. TLR activation of B cells could ultimately result in IgM secretion, which rapidly neutralizes pathogens, namely the TI antibody response (68). In this study, SUP3 directly promoted IgM production by B cells, suggesting its effects on both innate and adaptive immunity. Overall, SUP3 enhanced cellular immunity as well as TD and TI antibody production. This functional property of SUP3 made it a promising ideal adjuvant in immunotherapy and vaccination.

The more vigorous immune stimulatory activities of SUP3 compared to Pam3 in CTL induction, rejection of B16-OVA tumors, CD4^+^ T cell activation, and the production of TD antibodies could all attribute to its better stability and sustainable activity over Pam3 *in vivo*. Thus, SUP3 could act as an effective adjuvant in immunization.

An ideal adjuvant should have minimum or even none adverse effects. Generally, TLR agonists are potent immune stimuli capable of inducing large amount of inflammatory cytokines, which may lead to cytokine storm to damage the hosts before inducing protective immune responses. Our study and previous studies showed that engagement of TLR2 by triacyl lipopeptides did not promote severe inflammatory responses, which would be beneficial for its clinical applications (65, 69, 70). Ligation of TLR2 also activated PI3K–Akt–β-catenin axis to induce IL-10 expression, which is a regulatory cytokine to suppress inflammation (70). TLR2 agonists cloud also induce rapid degradation of IRAK1, which is an essential component of inflammatory response (71). These effects might contribute to the weaker inflammatory properties of TLR2 agonists. In addition, TLR2 ligation signal *via* adaptor MyD88 to promote downstream events, whereas LPS recognition by TLR4 can activate both MyD88 and TRIF adaptors mediated pathways (72, 73). Thus, LPS could induce stronger inflammatory responses *via* two pathways than that induced by TLR2 agonists. Meanwhile, this immune regulatory effect did not attenuate T cell activation by DCs (74). Again, SUP3 could be considered as a novel effective immune adjuvant.

Adjuvants are not only applied in vaccination but also in cancer immunotherapy. CTLs induced by DCs are major knights to combat tumors. Enhanced DC activation will lead to vigorous antitumor cytotoxicity by CTLs. Our studies proved that SUP3 conferred more protective immunity from tumor metastasis to the lungs. Tumors escape from immune attack by diminishing CTL responses. PD-1/PD-L1 is important check-point molecules during immune responses, which suppress antitumor immune responses. Blocking and eliminating their inhibitory effects by neutralizing antibodies significantly enhanced antitumor immune responses and had been used in clinical trials for cancer therapies (75–77). However, this treatment is not always effective for some patients due to various reasons. A combination of an effective immune adjuvant together with anti-PD-1/PD-L1 antibodies may improve the efficacy of the treatment. Our synthetic TLR2 agonist SUP3 could be an outstanding candidate for this purpose. Further tests are needed to determine the effectiveness of this strategy.

Overall, in this study, we developed a novel TLR2 agonist SUP3, which is more stable than Pam3 and could enhance DC functions without inducing excessive inflammation, the most favorable property of an ideal adjuvant. As DCs are essential initiators of antigen-specific immune responses, the enhanced activation and function of DCs by TLR2 agonists should improve immune responses to pathogens or tumors. Our study suggested that the new TLR2 agonist SUP3 could be a promising adjuvant in vaccination and immune modulations.

## Author Contributions

The study was designed by XG, NW, YS, JH, XBL, and LW. XG, XL (4th author), TW, YZ, XL (7th author) and JH preformed experiments and acquired the data. XG, NW, YS, XL (7th author), JH, XBL, and LW analyzed and interpreted the data. Manuscript was written by XG, NW, YS, XBL, and LW. This study was supervised by LW and XBL.

## Conflict of Interest Statement

The authors declare that the research was conducted in the absence of any commercial or financial relationships that could be construed as a potential conflict of interest.

## Abbreviations

CFSE, carboxyfluorescein diacetate, succinimidyl ester; CTL, cytotoxic T lymphocyte; DC, dendritic cell; GM-CSF, granulocyte-macrophage colony-stimulating factor; LPS, lipopolysaccharide; MAPK, mitogen-activated protein kinase; MHC, major histocompatibility complex; NP-KLH, nitrophenyl-keyhole limpet hemocyanin; OCS, OVA-coated splenocytes; OVA, ovalbumin; PBS, phosphate buffered saline; TD antigen, thymus-dependent antigen.
