# Supplementary material for: The Novel Toll-Like Receptor 2 Agonist SUP3 Enhances Antigen Presentation and T Cell Activation by Dendritic Cells
Source: Front Immunol. 2017 Feb 21;8:158. doi: 10.3389/fimmu.2017.00158 (PMC5318439; doi:10.3389/fimmu.2017.00158)
Supplement: Supplementary file 1 [file Presentation_1.PPTX]

## Slide 1
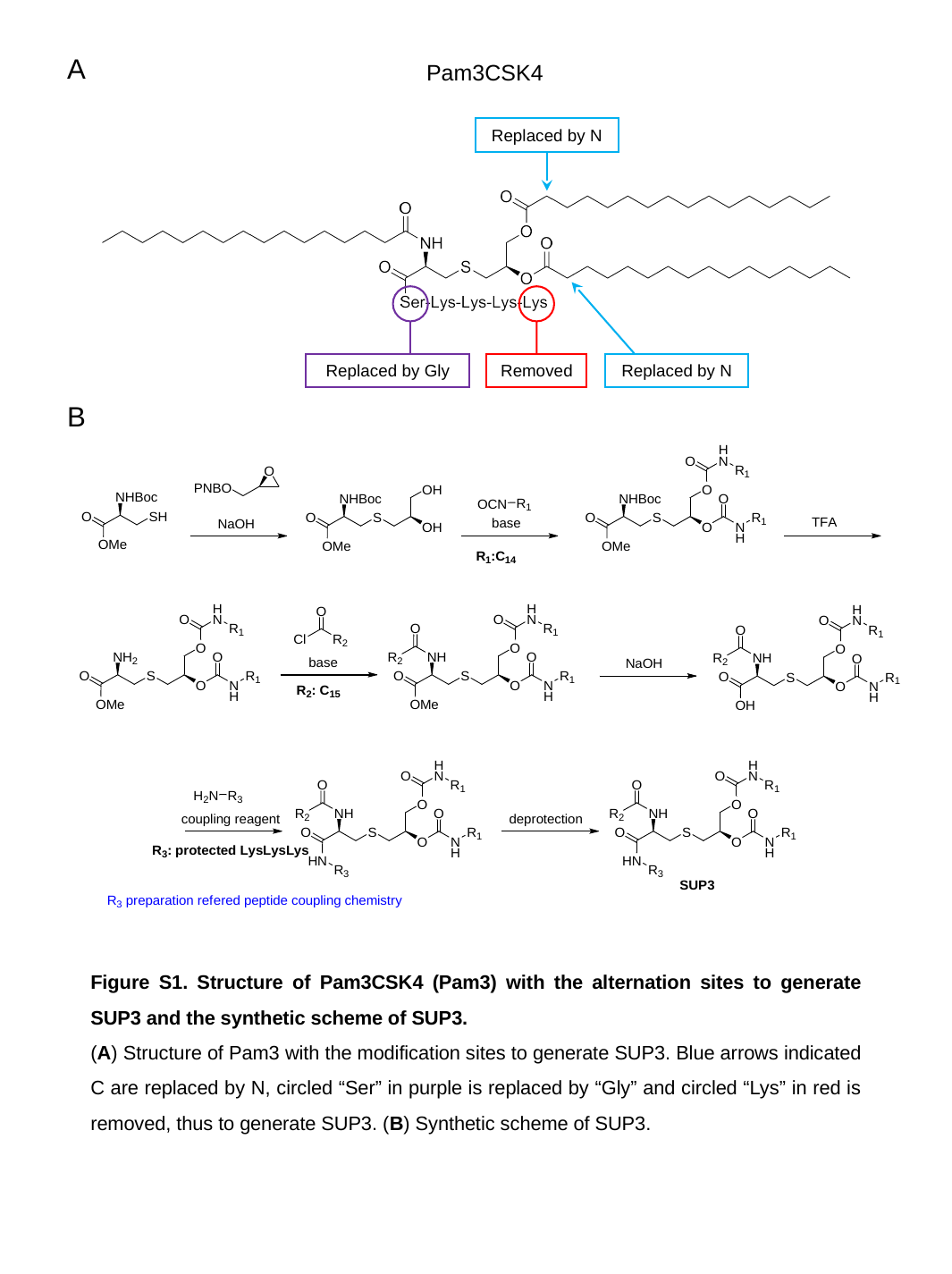

A
Pam3CSK4
Replaced by N
Replaced by Gly
Removed
Replaced by N
B
Figure S1. Structure of Pam3CSK4 (Pam3) with the alternation sites to generate SUP3 and the synthetic scheme of SUP3.
(A) Structure of Pam3 with the modification sites to generate SUP3. Blue arrows indicated C are replaced by N, circled “Ser” in purple is replaced by “Gly” and circled “Lys” in red is removed, thus to generate SUP3. (B) Synthetic scheme of SUP3.

## Slide 2
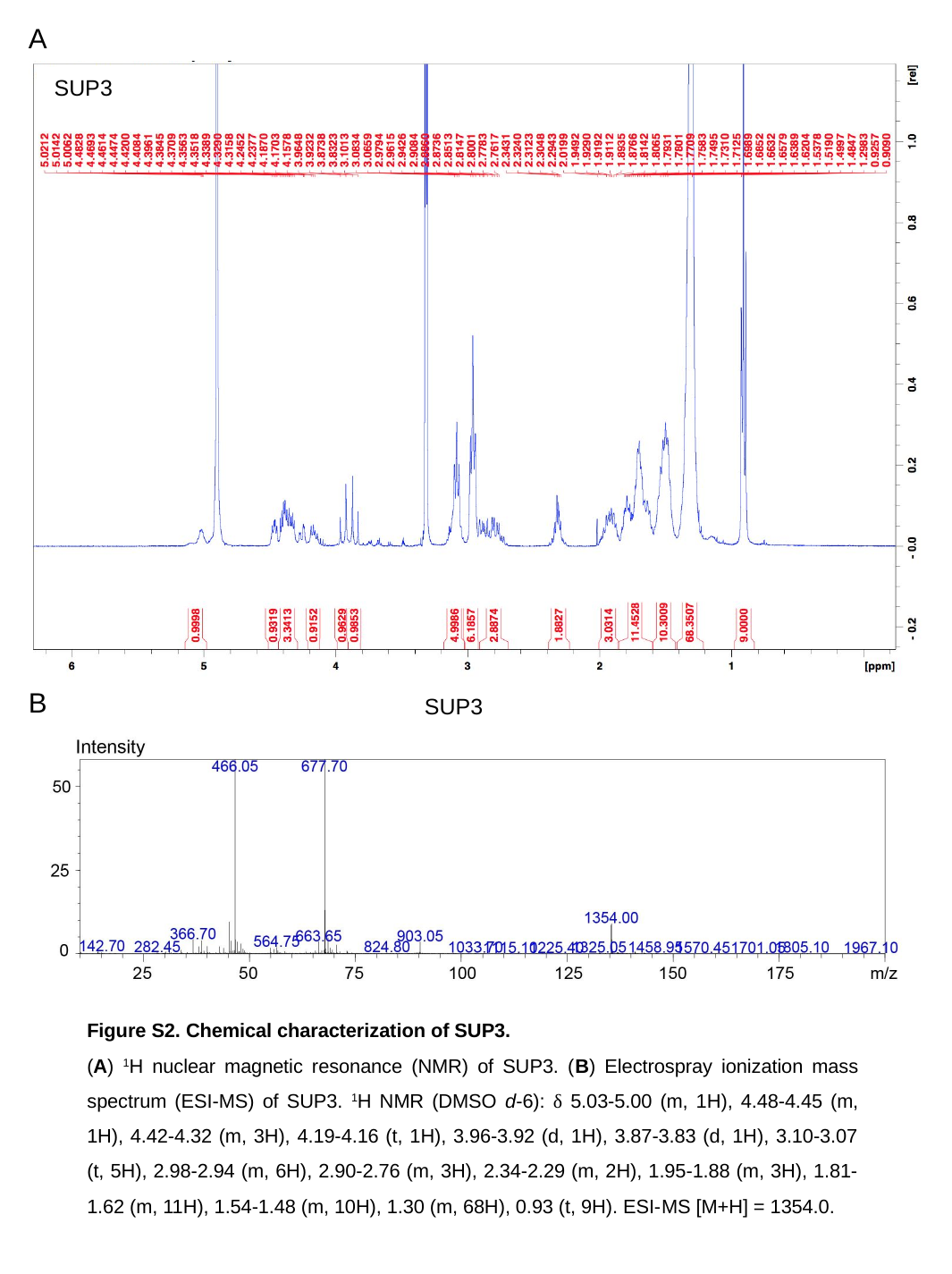

A
SUP3
B
SUP3
Figure S2. Chemical characterization of SUP3.
(A) 1H nuclear magnetic resonance (NMR) of SUP3. (B) Electrospray ionization mass spectrum (ESI-MS) of SUP3. 1H NMR (DMSO d-6): δ 5.03-5.00 (m, 1H), 4.48-4.45 (m, 1H), 4.42-4.32 (m, 3H), 4.19-4.16 (t, 1H), 3.96-3.92 (d, 1H), 3.87-3.83 (d, 1H), 3.10-3.07 (t, 5H), 2.98-2.94 (m, 6H), 2.90-2.76 (m, 3H), 2.34-2.29 (m, 2H), 1.95-1.88 (m, 3H), 1.81-1.62 (m, 11H), 1.54-1.48 (m, 10H), 1.30 (m, 68H), 0.93 (t, 9H). ESI-MS [M+H] = 1354.0.

## Slide 3
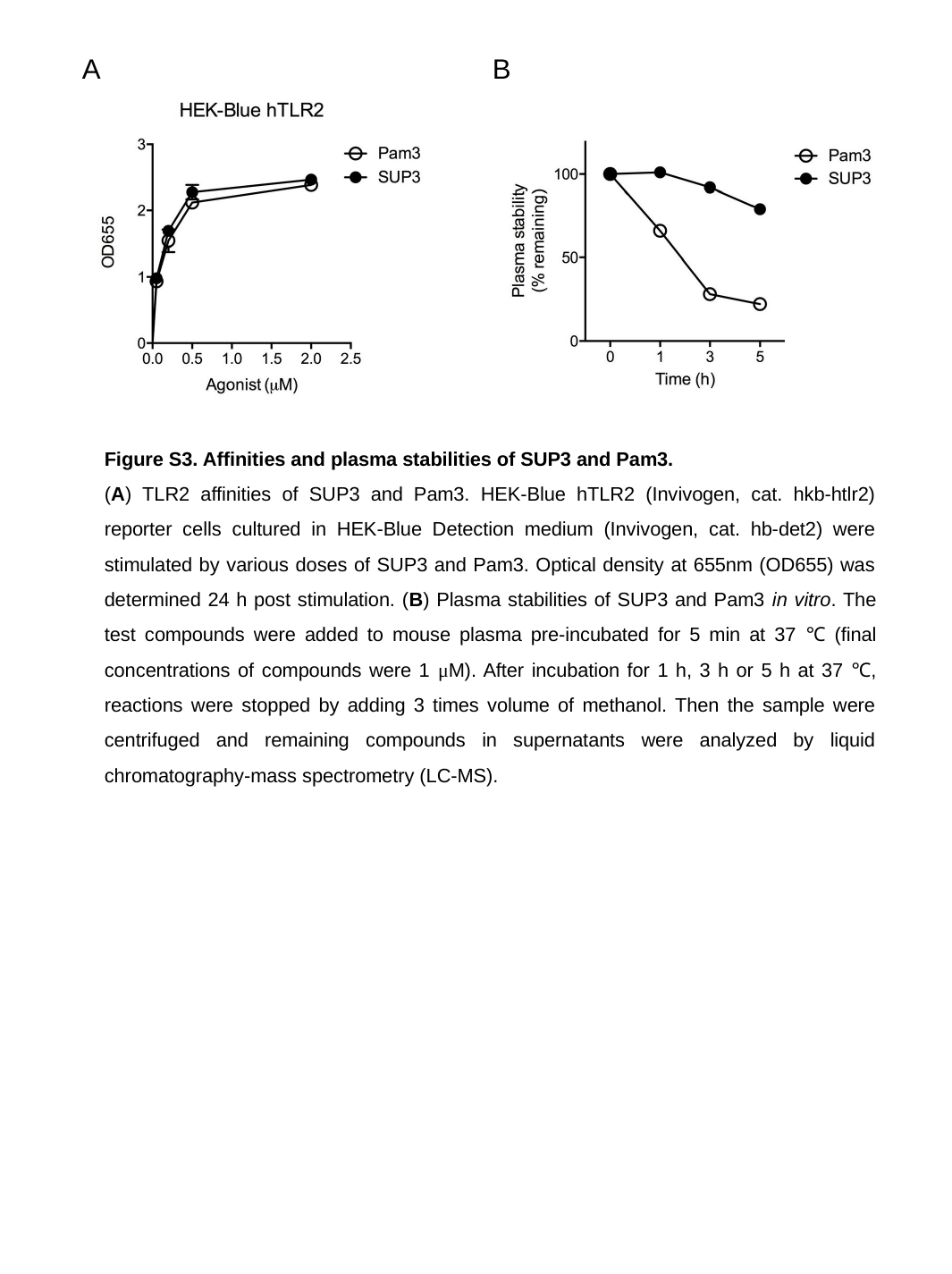

A
B
Figure S3. Affinities and plasma stabilities of SUP3 and Pam3.
(A) TLR2 affinities of SUP3 and Pam3. HEK-Blue hTLR2 (Invivogen, cat. hkb-htlr2) reporter cells cultured in HEK-Blue Detection medium (Invivogen, cat. hb-det2) were stimulated by various doses of SUP3 and Pam3. Optical density at 655nm (OD655) was determined 24 h post stimulation. (B) Plasma stabilities of SUP3 and Pam3 in vitro. The test compounds were added to mouse plasma pre-incubated for 5 min at 37 ℃ (final concentrations of compounds were 1 μM). After incubation for 1 h, 3 h or 5 h at 37 ℃, reactions were stopped by adding 3 times volume of methanol. Then the sample were centrifuged and remaining compounds in supernatants were analyzed by liquid chromatography-mass spectrometry (LC-MS).

## Slide 4
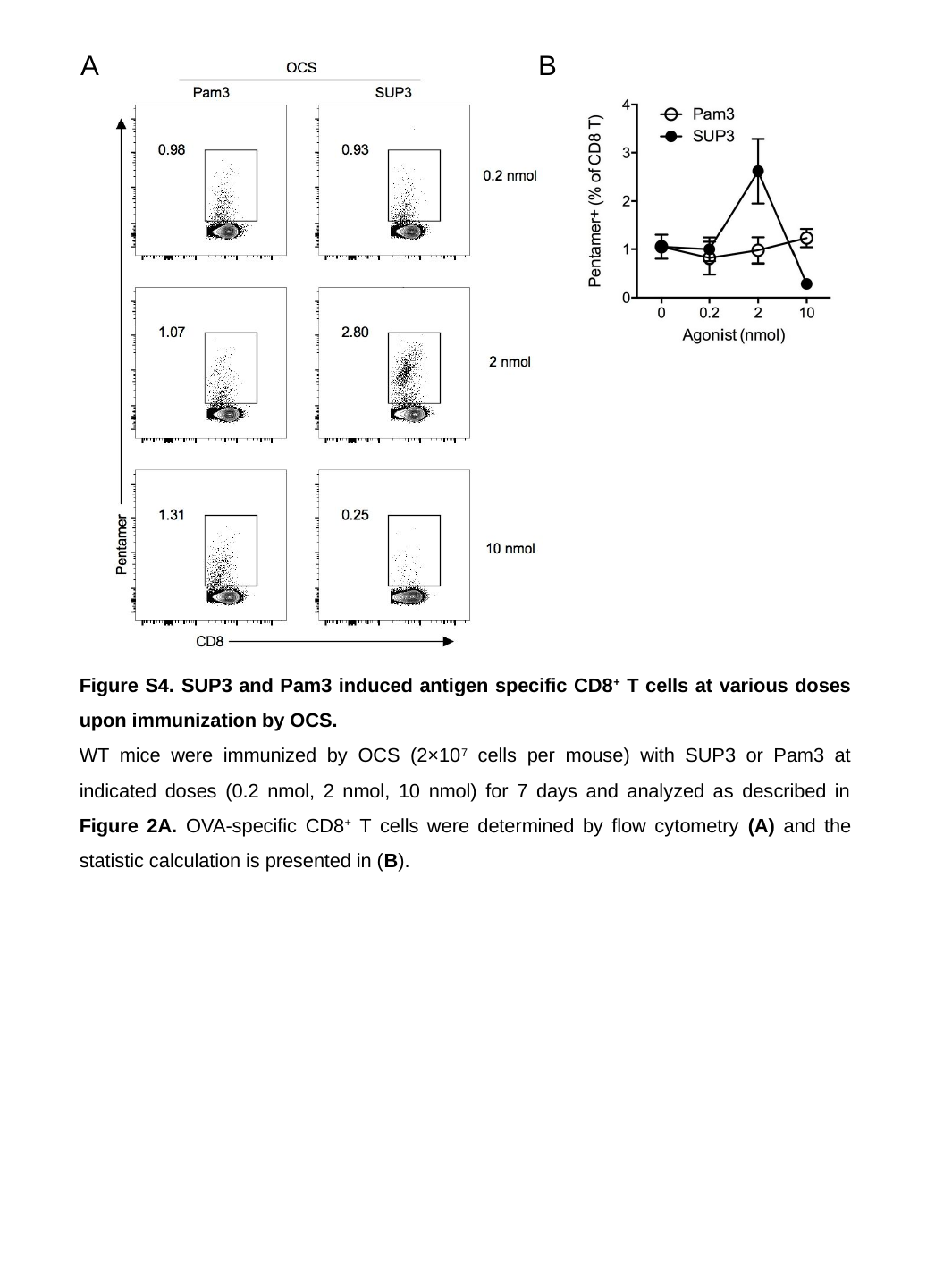

A
B
Figure S4. SUP3 and Pam3 induced antigen specific CD8+ T cells at various doses upon immunization by OCS.
WT mice were immunized by OCS (2×10^7^ cells per mouse) with SUP3 or Pam3 at indicated doses (0.2 nmol, 2 nmol, 10 nmol) for 7 days and analyzed as described in Figure 2A. OVA-specific CD8+ T cells were determined by flow cytometry (A) and the statistic calculation is presented in (B).

## Slide 5
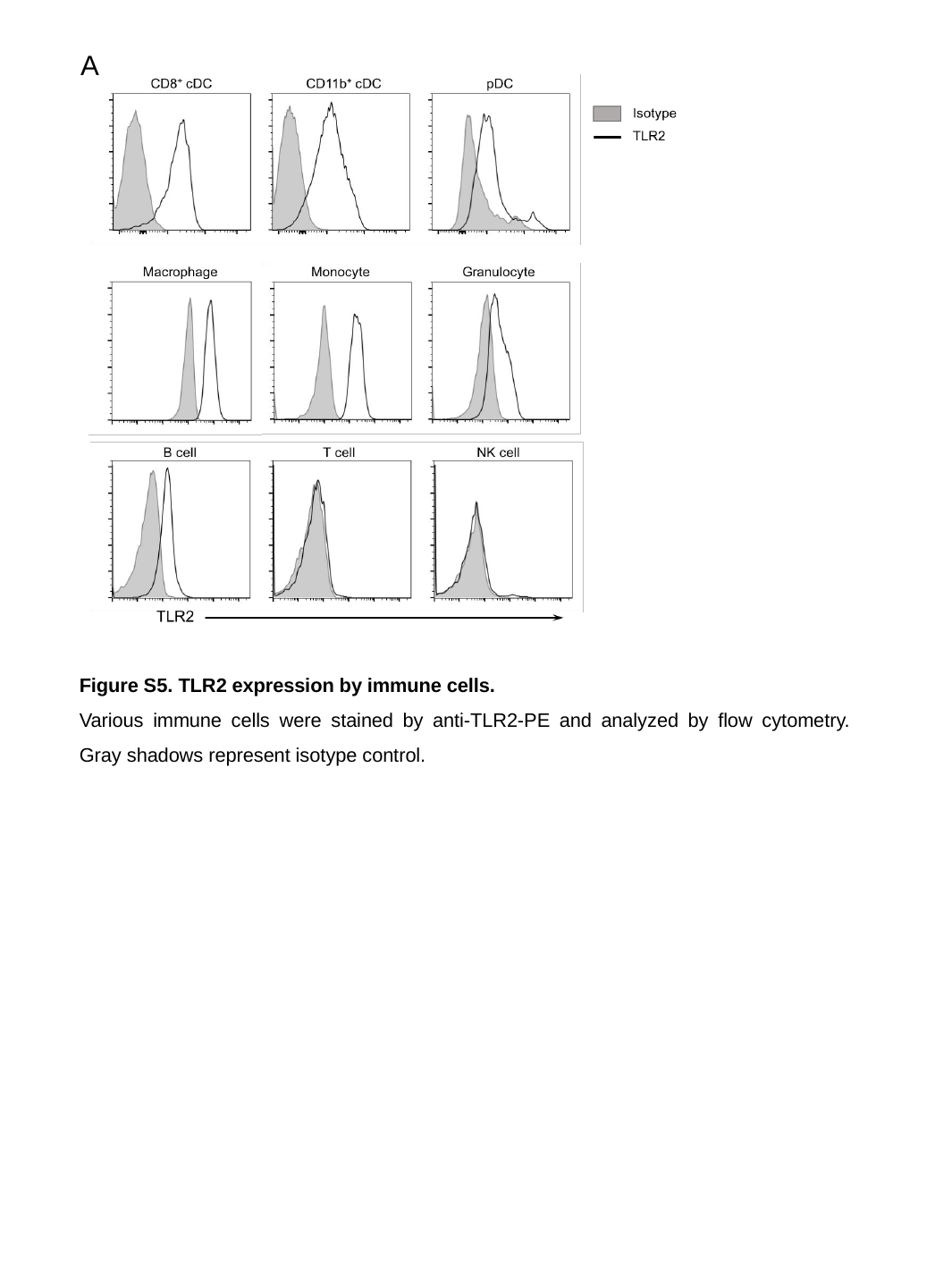

A
Figure S5. TLR2 expression by immune cells.
Various immune cells were stained by anti-TLR2-PE and analyzed by flow cytometry. Gray shadows represent isotype control.
